# Control and coding of pupil size by hypothalamic orexin neurons

**DOI:** 10.1038/s41593-023-01365-w

**Published:** 2023-06-19

**Authors:** Nikola Grujic, Alexander Tesmer, Ed Bracey, Daria Peleg-Raibstein, Denis Burdakov

**Affiliations:** Neurobehavioural Dynamics Laboratory, Department of Health Sciences and Technology, ETH Zurich, Schwerzenbach, Switzerland

**Keywords:** Neuroscience, Sensory processing, Biomarkers

## Abstract

Brain orexin (hypocretin) neurons are implicated in sleep–wake switching and reward-seeking but their roles in rapid arousal dynamics and reward perception are unclear. Here, cell-specific stimulation, deletion and in vivo recordings revealed strong correlative and causal links between pupil dilation—a quantitative arousal marker—and orexin cell activity. Coding of arousal and reward was distributed across orexin cells, indicating that they specialize in rapid, multiplexed communication of momentary arousal and reward states.

## Abstract (web summary)

The biological meaning of eye pupil size is a subject of intense research. This study shows that pupil fluctuations reveal information about hypothalamic orexin cells, which control pupil size via a noradrenaline neural circuit.

## Main

The orexin (hypocretin) system of the lateral hypothalamus (LH) projects brain-wide, with particularly strong connections to arousal and reward centers^1–3^. Through these connections, the orexin network regulates sleep–wake switching and autonomic function, as well as feeding and exploratory behaviors^4–9^. Pupil dilation is routinely used in human experiments as a measure of arousal and autonomic function^10^, and for predicting key aspects of cognition, such as the exploration–exploitation balance^11,12^. As such, and with its strong tracking of the locus coeruleus (LC) noradrenergic system^13,14^, pupil size measurements have been used to support the adaptive gain theory of arousal^12,15^. Pupil dilation has also been correlated to the activity of cholinergic^14^ and serotonergic^16^ neuromodulatory systems. However, orexinergic modulation of moment-to-moment arousal, as indexed by pupil dilation, has not been explored, and previous studies contain arguments for both constriction^17,18^ and dilation^19–22^. In particular, the related question of the interplay of arousal and reward representations in individual orexin neurons^9^ has not been answered experimentally.

**Fig. 1:**
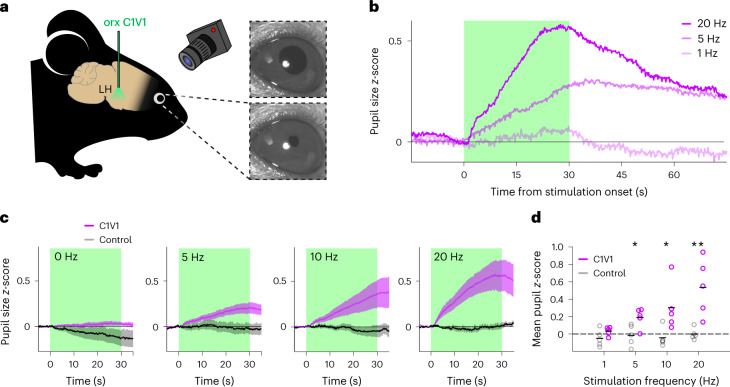
Orexin cell stimulation elicits pupil dilation. **a**, Mouse pupils were recorded under isoflurane anesthesia during optostimulation of orexin neurons in the LH. **b**, Example pupil responses of one mouse to different stimulating frequencies. The duration of stimulation is indicated by the green shaded area. **c**, Pupil responses for the opsin (*n* = 5 mice) and control (*n* = 5 mice) groups at increasing stimulation frequencies (left to right, the shaded areas show the s.e.m.). **d**, Mean pupil sizes for the last 10 s of stimulation; the asterisks indicate significant differences between groups (*n* = 5 mice in both experimental (C1V1) and control groups; two-tailed Mann–Whitney *U*-test, left to right frequencies: *U* = 6, 2, 2, 0, *P* = 0.22, *0.03, *0.03 and **0.008); the means are shown as overlay. Source data

To causally test the pupil dilation responses elicited by orexin cell activation, we selectively opto-stimulated LH orexin neurons, while tracking pupil diameter in anesthetized mice (Fig. 1a and Methods). We observed rapid dilation in response to the stimulation, which declined after stimulation offset (Fig. 1b–d, statistics are given in the figure legend). The effect was stimulation-frequency dependent, with higher frequency stimulation eliciting faster and greater pupil dilation (Fig. 1b–d).

**Fig. 2:**
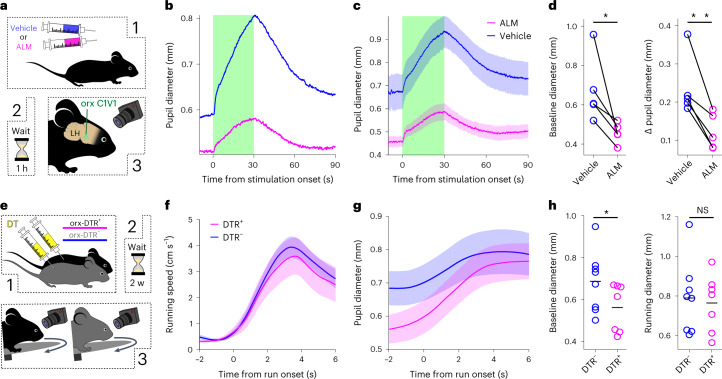
Disruption of orexin cell function leads to observable pupil size differences. **a**, The pupil was recorded during 20-Hz optostimulation of LH orexin neurons in ALM- or vehicle-injected, isoflurane anesthetized mice. **b**, An example pupil size trace from one mouse during orexin cell optostimulation after ALM or vehicle injection. The green shaded area indicates optostimulation. **c**, Absolute pupil size across mice (mean ± s.e.m. of *n* = 5 mice). **d**, Left, within mouse comparison of baseline pupil diameters after vehicle or ALM injections (*n* = 5 mice, one-tailed paired *t*-test, *t* = 2.8, *P* = 0.02). Right, within mouse comparison of change in pupil diameter from baseline to peak during laser stimulation (one-tailed paired *t*-test, *t* = 4.75, *P* = 0.0045). **e**, Pupil diameter of orx-DTR^+^ and orx-DTR^−^; DT-injected mice were recorded during head-fixed running on a wheel. **f**, Mean speed within *k*-means-identified clusters of equivalent running bouts from DTR^+^ and DTR^−^ mice (mean ± s.e.m. of *n* = 7 orx-DTR^+^ and *n* = 8 orx-DTR^−^ mice). **g**, Pupil dynamics (means ± s.e.m.) corresponding to the running bouts shown in **f**. **h**, Left, comparison of baseline pupil diameters (at −2 s from run onset) for DTR^−^ (*n* = 8) and DTR^+^ (*n* = 7) mice (one-tailed *t*-test, *t* = 1.8, *P* = 0.048). Right, comparison of pupil diameter during the run bout (at +6 s from run onset; one-tailed *t*-test, *t* = 0.3, *P* = 0.39). *P < 0.05; NS, not significant. Source data

**Extended Data Fig. 1:**
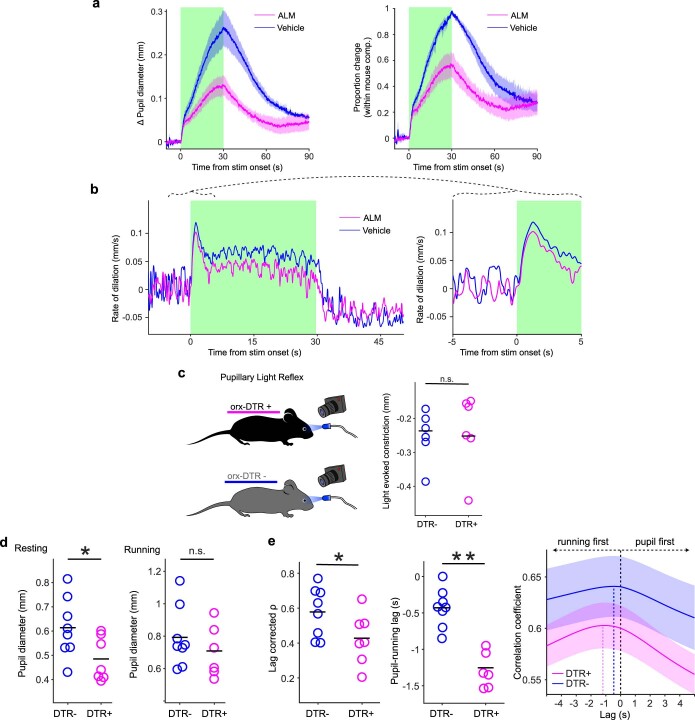
Additional analyses of pupil alterations upon orexin cell function disruption. **a)** Left: mean baseline-subtracted pupil size in same mice after vehicle or ALM injection. Right: mean pupil size normalized to peak dilation in control (vehicle injected) mice after vehicle or ALM injection. **b)** Dilation rates during orexin cell opto-stimulation and orexin receptor blockade. Mean rates of dilation across mice during 20 Hz opto-stimulation of orexin cells in the LH. Shaded green area indicates stimulation duration. Left, dilation rates for Almorexant-injected animals (pink) vs. same animals after vehicle injection (blue). Right, zooms of 5 seconds before and after stimulation onset. **c)** Left: Orexin-cell-ablated (DTR+) or wild-type (DTR-) mice were delivered 15 s long blue LED pulses to the eye while recording the pupil. Right: comparison of light-evoked constriction (baseline diameter to minimum diameter) between DTR+ and DTR- mice (one-tailed t-test, t = 0.28, p = 0.39, n = 6 DTR+ and 6 DTR- mice). **d)** Comparison of mean pupil diameters for DTR- and DTR+ mice over entire sessions. Left: during resting epochs (one-tailed t-test, t = 2.3, p = 0.02, n = 7 DTR+ and 8 DTR- mice). Right: during running epochs (one-tailed t-test, t = 0.95, p = 0.18, n = 7 DTR+ and 8 DTR- mice). **e)** Comparison of pupil-running synchrony over entire sessions. Left: comparison of lag-subtracted Spearman’s rank correlations coefficients between running speed and pupil size (one-tailed t-test, t = 2, p = 0.03, n = 7 DTR+ and 8 DTR- mice). Middle: mean pupil radius to running onset lag at maximum correlation values for the two groups (one-tailed t-test, t = 6.1, p = 0.00002, n = 7 DTR+ and 8 DTR- mice). Right: cross-correlation analysis of pupil radius and associated running signals. Mean correlation coefficients at different lags for the two groups. In all panels: shaded areas around traces indicate s.e.m. Source data

Next, we tested how disruption of orexin neuropeptide signaling affected pupil size in awake and anesthetized animals. To distinguish the contribution of orexin neurotransmission to pupil dilation from other neurotransmitters emitted by orexin neurons, we repeated the optogenetic stimulation experiment (Fig. 1) while specifically blocking orexin receptors with the antagonist almorexant (ALM) (Fig. 2a and Methods). ALM reduced both tonic pupil dilation (Fig. 2b–d) and the extent of pupil dilation during the orexin cell optostimulation-evoked response (Fig. 2b–d and Extended Data Fig. 1a). Interestingly, rapid dilation at optostimulation onset was initially similar between the ALM and vehicle conditions, but diverged after 1–2 s, with slower and smaller dilation occurring in ALM-injected mice thereafter (Extended Data Fig. 1b). This suggests that fast transmitter(s) released by orexin cells^23^ caused the initial dilation. Overall, these results establish a causal link between orexin neurotransmission and pupil size.

To investigate the effect of orexin neurons themselves on pupil modulation, we selectively ablated them using the orexin cell targeted diphtheria toxin receptor (DTR) mouse model (Methods). We first probed the role of orexin cells in light-induced pupil constriction (a possibility suggested by recent reports^17,18^), but found it similar in orexin-cell-ablated and control mice (Extended Data Fig. 1c). We then compared the pupil dynamics of orexin-cell-ablated and control mice, head-fixed but allowed to run freely on a wheel (Fig. 2e), and analyzed locomotion-state-related pupil dynamics. To avoid effects due to any running bout distortion in orexin-cell-ablated mice^7^, we analyzed the pupil dynamics associated with similar running bouts in control and orexin-cell-ablated mice (Fig. 2f,g). We found that pupil size was significantly reduced in orexin-cell-ablated mice during resting (Fig. 2h, left). However, during running, the differences in pupil size were not significant (Fig. 2h, right). The same findings were obtained when the entire recording session was considered (Extended Data Fig. 1d), where we also observed differences in pupil-running coupling (Extended Data Fig. 1e). This result shows that orexin neurons are necessary for normal control of pupil size in behaving animals, especially during resting epochs.

**Fig. 3:**
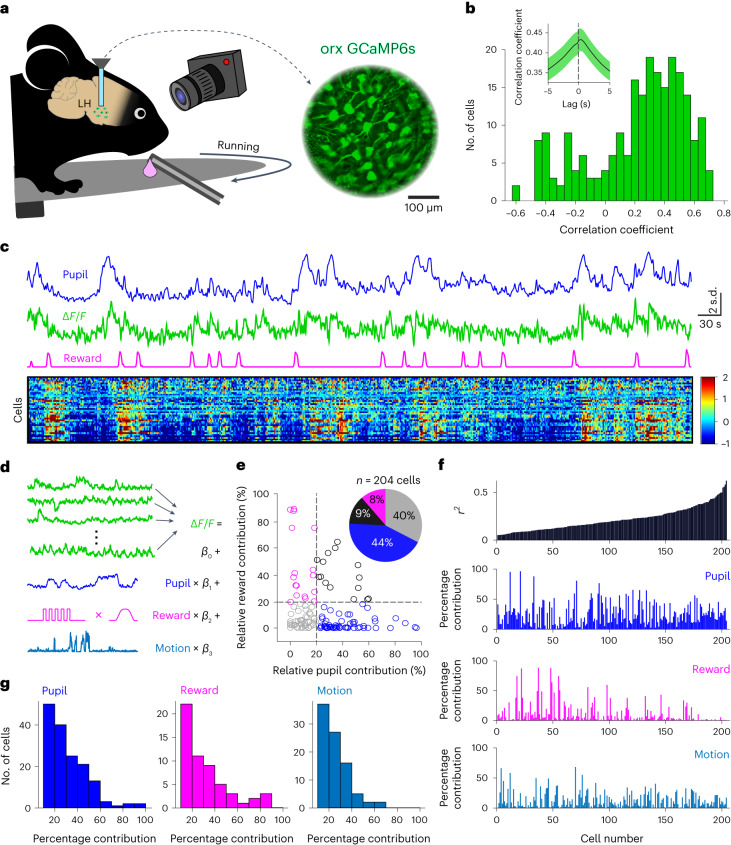
Coding of pupil size and other variables in individual orexin cells. **a**, Pupil was recorded during orx-GcaMP6s, two-photon, GRIN lens imaging of orexin neurons (*n* = 228 cells from 5 mice) during free running and reward consumption. **b**, The distribution of cell activity correlations with pupil size (*n* = 204 cells; cells with *r*^2^ < 0.05 were removed from the analyses; the inset shows cross-correlation of pupil ON cells from **c**, *n* = 42 cells, the shaded area is the s.e.m.). **c**, Time-aligned activity traces from one mouse. Top to bottom, pupil size, orexin neuron activity, convolved licking trace and a heatmap of activity of all ON-type orexin neurons in the session. **d**, The encoding model. Linear regression was used to quantify the linear relationships between measured variables and the activity of each cell separately. **e**, Scatterplot of relative contributions of pupil size and reward consumption to explained variance in each cell’s activity (each point is a separate cell). The pie chart shows the distribution with a 20% contribution cutoff. **f**, Top to bottom, the explained variance of each cell’s activity in a model encompassing all investigated variables, pupil, reward and locomotion percentage contributions to the explained variance. **g**, Distributions of cell contributions for each of the investigated variables showing only cells with more than 10% contribution. Source data

**Extended Data Fig. 2:**
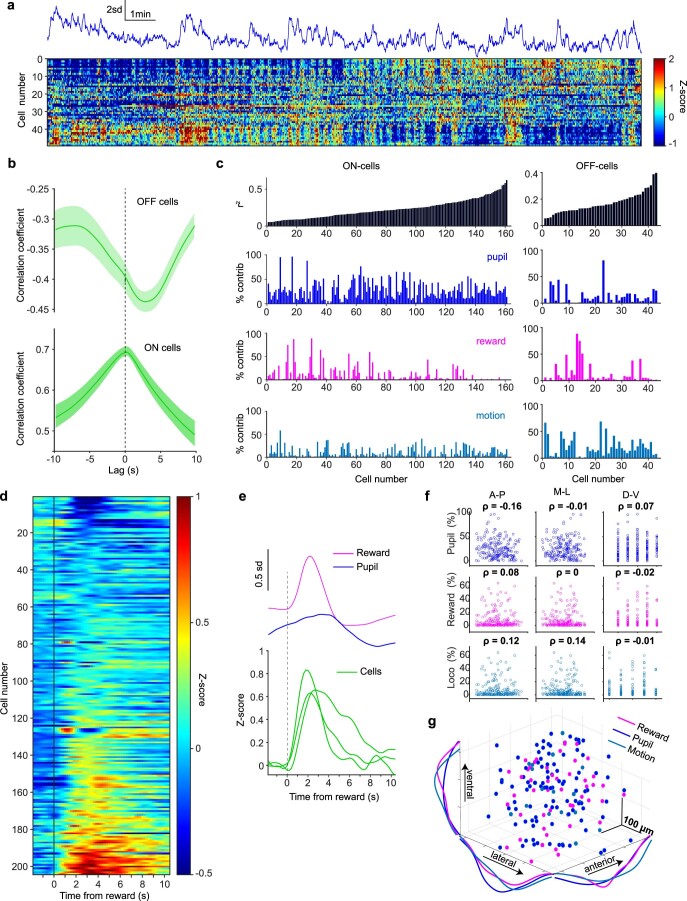
Additional analyses of volumetric GRIN 2 P data. **a)** Top, z-scored pupil trace aligned to (bottom), heatmap of orexin neuron activity arranged by correlation with pupil size from one mouse. **b)** Average of cross-correlations of pupil with cells positively correlated with pupil (ON-cells, bottom, n = 161 cells) or negatively correlated with pupil (OFF-cells, top, n = 43 cells). Shaded areas show s.e.m. **c)** R-squared values and contributions to variance explained in analysis shown in Fig. 3. Separated for ON-cells (left) and OFF-cells (right). **d)** Reward onset aligned, trial-averaged and baseline subtracted orexin cell activity for experiment shown in Fig. 3. **e)** Top, example pupil and splined licking trace from one animal. Bottom, reward-onset aligned traces from three orexin cells shown in heatplot in a. **f)** Spearman’s rank correlations between the three spatial dimensions of recorded cells and contributions of different variables to cell activity. **g)** Three-dimensional scatter-plot of cells sorted into groups of over 20% contribution of a given investigated variable to the cell’s activity. The projections on the axes show the fitted density distributions of the cells in each of the three dimensions. Source data

**Extended Data Fig. 3:**
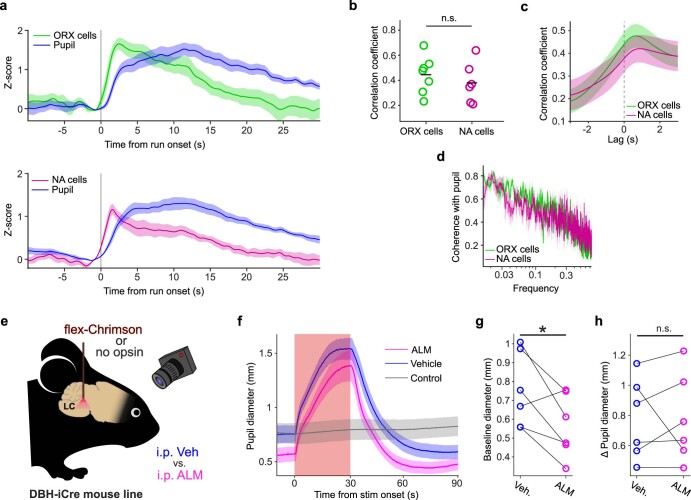
Additional data comparing LC and orexin neurons, and effect of orexin antagonism on LC pupil control. **a)** Run onset aligned traces of pupil and either orexin cells (ORX, top panel, n = 7 mice) or LC noradrenaline cells (NA, bottom panel, n = 6 mice) photometry signals. Shaded regions depict s.e.m. **b)** Lag corrected correlation coefficients between pupil size and orexin/LC neuron activity (n = 7 mice in ORX and n = 6 mice in LC recorded group, one-tailed t-test: t = −0.76, p = 0.23). **c)** Cross-correlation plot between pupil and respective neural signal. Shaded regions depict s.e.m. **d)** Coherence analysis between pupil and respective neural signals. **e)** Schematic for f-h: Mouse pupils were recorded under isoflurane anesthesia during LC opto-stimulation in mice whose LC noradrenaline neurons expressed flex-Chrimson or in control mice without any virus expression. **f)** Absolute pupil diameter (means±s.e.m.) during LC opto-stimulation. **g)** Within mouse comparison of baseline pupil diameters after vehicle or ALM injections (one-tailed paired t-test, t = 2.7, p = 0.02, n = 6 mice). **h)** Within mouse comparison of change in pupil diameter from baseline to peak during laser stimulation (one-tailed paired t-test, t = 0.02, p = 0.5, n = 6 mice). Source data

Orexin cell activity covaries with multiple factors, including reward and locomotion^7,9^. To investigate pupil size coding in individual orexin cells in relation to these variables in behaving mice, we used volumetric two-photon gradient-index (GRIN) lens imaging in LH. We monitored orexin-cell-targeted GCaMP6s activity, while the mouse was freely running on a wheel and receiving milkshake rewards at random intervals (Fig. 3a). We found that pupil size followed orexin cell activity closely, with net cell activation preceding dilation (Fig. 3b,c and Extended Data Fig. 3a,c). Individual cells were either positively or negatively correlated with pupil size (pupil ON and pupil OFF cells respectively, Fig. 3b). These cell types were pooled for further analysis because no obvious differences were found in the way they coded for the other investigated variables (Extended Data Fig. 2b,c). We also found cells whose activation was associated with reward consumption (Extended Data Fig. 2d,e). To quantify and compare the contribution of pupil size, locomotion and reward consumption to individual orexin cell responses, we used an encoding model based on multivariate linear regressions^24^ (Fig. 3d). By removing each of the predictors from the encoding model for each cell, and comparing the resulting *r*^2^ with the complete model, we could infer contributions of each predictor to the explained variance of that cell’s activity (Fig. 3e,f). This revealed distributed coding of pupil size and reward across orexin neurons. Some cells coded for both pupil and reward, but other cells represented only one of these variables, with a larger proportion of cells coding purely for pupil size with little reward contribution (Fig. 3e). This is in line with the predictions about the dichotomy of orexin function, representing both arousal and reward^9^. The cells’ coding properties were largely independent of their spatial locations (Extended Data Fig. 2f,g). Within the investigated variables, pupil size contributed most to explained variance in cell activity (Fig. 3e–g). These results establish pupil size as a strong readout of orexin activity and display how rapid representations of arousal, reward and movement are distributed across orexin neurons.

**Extended Data Fig. 4:**
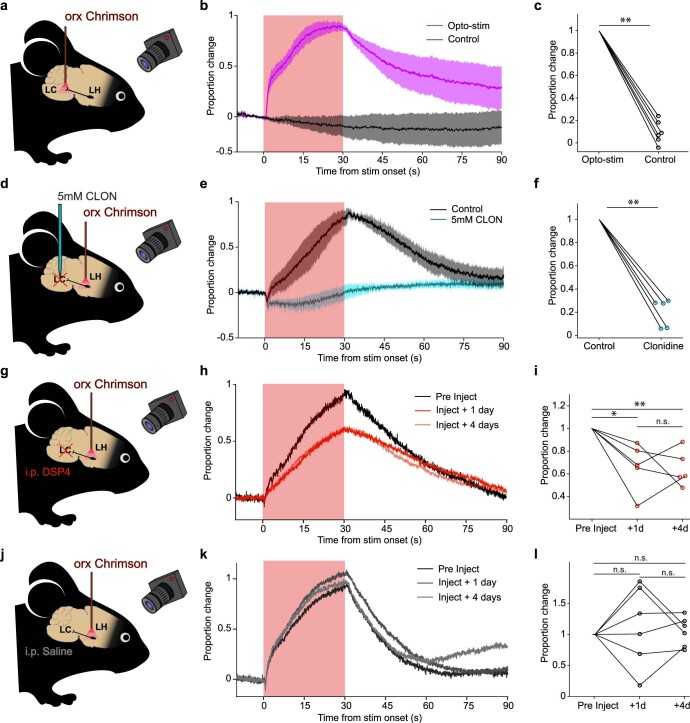
Role of LC in pupil control by orexin neurons. **а)** Schematic for data in b,c: orexin-ChrimsonR - expressing axons in LC selectively opto-stimulated (20 Hz) in isoflurane anesthetized animals. **b)** Pupil responses in control (no stimulation, gray) and opto-stimulation (magenta) trials (means ± s.e.m. of n = 6 mice), red-shaded area in this and other panels marks opto-stimulation. Data are normalized to sd-baseline and maximum opto-stimulation-evoked dilation per mouse. Non-opsin LC opto-stimulation control is shown in Extended Data Fig. 3f (control). **c)** Comparison of data in b at peak dilation (one-tailed paired t-test, t = −21.8, p = 0.000002). **d)** Schematic for data in e,f: LH orexin cells were opto-stimulated at 20 Hz after vehicle or 5 mM Clonidine injection into the LC. **e)** Pupil responses to orexin cell optostimulation after vehicle (gray) or clonidine (light blue) injection into the LC. Data are normalized to sd-baseline and maximum opto-stimulation-evoked dilation after vehicle injection per mouse. Data are mean ± s.e.m. of n = 5 mice. **f)** Comparison of data in e at peak dilation (one-tailed paired t-test, t = 14.6, p = 0.00012). **g)** Schematic for data in h,i: LH orexin cells were opto-stimulated at 20 Hz before and after i.p. injection DSP4 (100 mg/kg). **h)** Mean pupil responses (n = 5 mice) before DSP4 injection (black) and 1 and 4 days after injection (red). Data are normalized to sd-baseline and stimulation-peak of pre-injection day per mouse. **i)** Comparison of data in h at peak dilation (one-tailed paired t-tests: pre vs. +1d, t = 3.5, p = 0.012; pre vs. +4d, t = 4.9, p = 0.0039; +1d vs. +4d, t = 0.13, p = 0.55). **j)** Schematic for data in k,l: LH orexin cells were opto-stimulated at 20 Hz before and after saline injection, as control for the DSP4 experiment. **k)** Mean pupil responses (n = 5 mice) before saline injection (black) and 1 and 4 days after injection (red shades). Data are normalized to sd-baseline and stimulation-peak of pre-injection day per mouse. **l)** Comparison of data in k at peak pupil dilation (one-tailed paired t-tests: pre vs. +1d, t = 0.5, p = 0.68; pre vs. +4d, t = 0.47, p = 0.67; +1d vs. +4d, t = 0.4, p = 0.65). Source data

To compare our findings in the orexin system with the noradrenergic system, which is more conventionally associated with pupil dilation, we also investigated pupil tracking of either orexin or LC noradrenaline neuron activity using population-level fiber photometry in behaving mice. We found strong correlations between pupil and both LC noradrenaline and orexin neural activity, with no difference in correlation strength (Extended Data Fig. 3a–c). There was little difference in coherence between the noradrenaline or orexin cell activity and pupil size, with strong coherence at frequencies less than 0.1 Hz, similar to results previously reported for LC noradrenergic neurons^14^ (Extended Data Fig. 3d). Given these findings and the previous literature on the impact of orexin cells on the LC^19,22^, as well as potential reciprocal signaling from the LC to orexin cells^25,26^, we examined roles of the LC–orexin cell interactions in pupil control. Pupil dilation evoked by optostimulation of LC noradrenaline cells was not affected by orexin receptor antagonism (Extended Data Fig. 3e–h) arguing against a major involvement of an LC→orexin link. In relation to orexin→LC functional projection^19,22^, we found that stimulation of orexin cell axons in LC dilated the pupil (Extended Data Fig. 4a–c), while manipulations reported to suppress LC noradrenaline cell function (clonidine and DSP4; Methods) suppressed the effect of orexin cell body stimulation on pupil dilation (Extended Data Fig. 4d–l). This implies that orexin cell→LC signaling shapes orexin cells’ impact on the pupil.

In summary, we found strong causative and correlative evidence implicating the orexinergic system in the control of pupil dilation. Our data reveal both tonic and phasic effects of orexin cell activity on pupil size. Orexin cell activation promoted rapid pupil dilation, and LC was an important mediator of this effect. Orexin neurotransmission was important for increasing pupil dilation; it is likely that an orexin-cell-derived fast transmitter (probably glutamate^23^) was responsible for rapid-onset effects. Deletion of orexin neurons caused the pupil to be more constricted during rest but not running, suggesting a role for other neural systems in maintaining pupil dilation during locomotion. Most individual orexin neurons strongly correlated with pupil size and displayed distributed coding of pupil size, reward and locomotion. These results shed light on the dichotomy between arousal and reward coding in the orexin system^9^, showing that both can be represented in the same neurons, with the extent of each representation varying between neurons. In future work, it will be important to probe this across diverse ethologically relevant behaviors, where other influences such as vestibular inputs have a role.

As we showed that both LC noradrenaline and orexin cell activities were similarly related to pupil size, it will be important to investigate whether some functions previously attributed to LC based on pupil measurements could also involve orexin cell activity, such as arousal gain control^12,15^. Orexin neurons could partially mediate increases in arousal gain associated with exploratory behavior, and the effect of reward consumption on orexin neurons could be key for transitioning along the exploration–exploitation axis. Furthermore, slow metabolic state signaling to orexin neurons via nutrients and hormones^27–29^ would place orexin neurons as a centerpiece in mediating these transitions during, for example, foraging behaviors. The implications of the present results could also be explored for diagnosing orexin cell loss in multiple neurological disorders^30^.

## Methods

### Animals and surgery

All animal experiments were performed in accordance with the Animal Welfare Ordinance (TSchV 455.1) of the Swiss Federal Food Safety and Veterinary Office, and approved by the Zurich Cantonal Veterinary Office. Adult C57BL/6 mice (at least 8 weeks old) were used for experiments. The experiments involved only male mice, except for two plots (Fig. 2h, two females in the DTR^+^ dataset and three females in the DTR^−^ dataset; Extended Data Fig. 3g,h, three females); no differences between males and females were noted in these datasets and so the sexes were pooled in these figures. Mice were kept on a reversed 12 h–12 h light–dark cycle in temperature- and humidity-controlled rooms (22 ± 1 °C, 55 ± 5%, respectively); all experiments were performed during the dark phase.

To specifically target orexin neurons for stimulation or recordings, we used orexin promoter (hORX) vectors previously established to selectively target orexin neurons^7,31,32^, namely for optogenetics: AAV1-hORX-C1V1(t/t)-TS-mCherry (>10^13^ GC ml^−1^, Vigene Biosciences) or AAV9-hORX-ChrimsonR-mCherry (2 × 10^12^ GC ml^−1^, UZH Viral Vector Facility); and for calcium recordings: AAV1-hORX-GCaMP6s.hGH (2.5 × 10^12^ GC ml^−1^, Vigene Biosciences). The specificities of these viral constructs for orexin neurons were verified with histological analyses after the experiments^7,31,32^.

For surgeries, mice were anesthetized with 5% isoflurane, after which they were transferred to the stereotaxic surgery setup and maintained on 1.5–2% isoflurane. They were given analgesic, and lidocaine was applied to the scalp. An incision was made to access the cranium, and a small hole was drilled above the LH over each hemisphere (0.9 mm lateral and 1.4 mm posterior from bregma). In the case of GRIN lens implants, only one larger (0.5-mm radius) circular craniotomy was performed unilaterally.

The LH was injected at 5.4 mm depth from bregma with either 200 nl of AAV1-hORX.C1V1(t/s).mCherry or AAV9-hORX-ChrimsonR-mCherry for the stimulation experiments, or with 300 nl AAV1-hORX.GCaMP6s for photometry, two-photon recordings, as well as controls for the channelrhodopsin stimulation experiments (where GCaMP served as a non-opsin control virus as in previous work^7^). Either two optic fibers (0.2 mm, Thorlabs) or a GRIN lens (0.6 mm, Inscopix) was lowered to the injection locations and cemented in place along with a custom-made headplate (Protolabs).

To specifically target noradrenaline cells in the LC, AAV9.CAG.Flex.GCaMP6s.WPRE.SV40 (1.9 × 10^13^ GC ml^−1^, Addgene) was used for photometry; AAV9-EF1a-DIO-ChrimsonR-mRuby2-KV2.1-WPRE-SV40 (0.5 × 10^12^ vg ml^−1^) was used for optostimulation experiments. Injections were performed in the LC of the previously validated C57BL/6-Tg(Dbh-icre)1Gsc mice (MGI ID 4355551, *n* = 6 for photometry and *n* = 6 for stimulation). Mice were injected unilaterally with 200 nl at depths of −3.5 and −3.7 mm, 0.9 mm lateral and 5.4 mm posterior from bregma. A fiber was implanted and cemented in place at −3.4 mm. For the experiments targeting orexin neuron projections to the LC (*n* = 6 mice), we implanted the optic fiber for stimulation at the above LC coordinates, while we injected the LH with AAV9-hORX-ChrimsonR-mCherry as also described above.

Animals were allowed a minimum of 3 weeks to express the viruses after injection before the experiments started.

### Pupil size measurement

In all experiments, pupils were recorded using an infrared camera (Blackfly FLIR, Spinnaker SDK program) at a frame rate of 20 Hz. Pupil size was determined by finding point estimates of eight points at the edge of the pupil in each frame, using DeepLabCut^33^. During blinking or low-confidence estimation of points, points were interpolated. A circle was then fitted to the estimated points in MATLAB to determine pupil surface area and radius.

### Optogenetic stimulation

Optogenetic stimulation of orexin neurons was performed using green (532 nm) or red (635 nm) lasers (Laserglow) for C1V1 and ChrimsonR, respectively via a 0.2-mm diameter optic fiber. Light intensity measured at the fiber end was 10 mW, and the 5-ms pulses were delivered at different frequencies, as specified in the figure legends. In Fig. 1, the animals received a 30-s optogenetic stimulation train at either 1, 5, 10 or 20 Hz, in a randomized order, every 2–2.5 min. In all other stimulation experiments, a 20-Hz, 30-s optogenetic stimulation train was delivered every 2–2.5 min.

### Pharmacological experiments

Orexin receptor blockade was performed with 100 mg kg^−1^ intraperitoneal injections of the dual orexin receptor antagonist Almorexant (ALM) dissolved in 10% dimethylsulfoxide and PBS vehicle. Mice were injected with either ALM or vehicle on separate days, 1 h before anesthetizing and performing optical stimulation. Stimulation was performed as described above.

To suppress LC function, we used two manipulations with different modes of action: local, stereotaxic-guided LC infusion of clonidine and systemic injection of the LC norepinephrine-depleting toxin DSP4 (*N*-(2-chloroethyl)-*N*-ethyl-2-bromobenzylamine hydrochloride). The rationale for this was based on previous publications showing that clonidine suppresses LC noradrenergic neurons by activating the inhibitory α2-adrenoceptor^34–37^, while DSP4 treatment selectively degrades the LC noradrenergic function to 10–30% of its normal value^38^. In the clonidine experiments (Extended Data Fig. 4d–f), we locally infused into the LC (same coordinates as stated above) 600 nl of 5 mM clonidine dissolved in saline, or 600 nl saline control, based on McBurney-Lin et al.^37^. In the DSP4 experiments (Extended Data Fig. 4g–i), we intraperitoneally injected 100 mg kg^−1^ of DSP4 or saline (Extended Data Fig. 4j–l), based on Ross and Stenfors^38^.

### Studies of mice without orexin neurons

For complete ablation of orexin neurons, we used mice expressing human DTR in orexin cells (orx-DTR mice); diphtheria toxin (DT) injection in this mouse model produces complete orexin cell loss while sparing surrounding cell types, as previously described and validated^31^. Orexin neurons were deleted in orx-DTR mice with two intraperitoneal injections of 150 ng DTR toxin (catalog no. D0564, Sigma-Aldrich) diluted to 1 μg ml^−1^ in saline, 2 d apart. Wild-type mice, used as controls, were DT-injected and analyzed in the same manner. After allowing 2 weeks to produce complete orexin deletion^31^, each mouse underwent two to four pupil recording sessions (30 min to 1 h each), performed on separate days. During recording, the animal was head-fixed to a custom-made post and allowed to run freely on a rotating wheel with a diameter of 20 cm. Running speed was captured using a rotary encoder attached to the wheel, and the pupil was recorded at 20 frames per second using a Blackfly FLIR camera. To select equivalent running bouts in each of the control and orexin-cell-ablated mice (Fig. 2f), we used *k*-means clustering on *z*-scored bouts. Clusters were identified using Lloyd’s algorithm with a maximum of 1,000 iterations.

In an alternative analysis (Extended Data Fig. 1d), we analyzed pupil size during both running and resting for the entire recording session using a binary threshold set at less than 1 cm s^−1^ or greater than 1 cm s^−1^, respectively. To investigate the effect of orexin neuron deletion on the pupillary light reflex (Extended Data Fig. 1c), we recorded the pupil in anesthetized, orexin-cell-ablated or control mice as we flashed a blue LED light at the contralateral eye for 15 s every 2 min.

### Volumetric two-photon imaging of single orexin neurons

Imaging was performed as described in our previous work^7^, using a custom electro-tunable lens-equipped resonant/galvanometer scan head two-photon microscope (INSS) and a femtosecond-pulsed, mode-locked Ti-sapphire laser (Spectra-physics Mai Tai HP Deepsee 2) at 950 nm through a 20× (0.45 numerical aperture, Olympus) air-IR objective at 31 frames per second. Custom Labview software was used to capture 512 × 512 pixel images of neurons through the implanted GRIN lens and a 510/80-nm band-pass emission filter. Six Z-planes were imaged with the electro-tunable lens, leading to a volume rate of 5.15 volumes per second.

During the imaging of orexin neurons in the LH, mice were head-fixed, allowed to run freely on a wheel and given 5-μl rewards of strawberry milkshake through a spout placed by their mouth. Rewards were delivered in random intervals of 1–1.5 min, with a total of 50 rewards delivered in a session, using a solenoid valve (catalog no. 161K011, NResearch). The metal reward spout was connected to a custom-made capacitive lick detector (catalog no. SEN-14520, Sparkfun Electronics). Because the pupil diameter in awake mice was too dilated to monitor in the dark, we elicited mild constriction with a constant weak blue LED light directed at the eye. To simultaneously track pupil size and accurately synchronize signals, a Blackfly FLIR camera frame capture was synced to a single plane capture of the two-photon microscope.

Acquired images were first motion-corrected using the TurboReg plug-in for ImageJ (National Institutes of Health). Regions of interest (ROIs) were drawn around each cell manually in ImageJ, and the mean intensity was extracted from each ROI to get the raw fluorescence of each cell. To correct for neuropil contamination, we also extracted the mean intensity of a halo surrounding each cell ROI with a distance of 6–12 pixels, but not including other ROIs. Finally, to get the final Δ*F/F* signal we subtracted the mean neuropil ROI intensity from the cell ROI intensity. Each Z-plane in the volume was inspected to find cells appearing in multiple planes; Δ*F/F* traces belonging to the same cell were averaged together. Each Δ*F/F* trace was smoothed with a three-sample moving average and *z*-scored. The collected dataset consisted of Δ*F/F* traces from 228 neurons (between 37 and 62 per mouse, *n* = 5 mice).

### Encoding model

The applied encoding model was based on a previous study^24^, and involved multiple regressions with the Δ*F/F* trace of each cell as a dependent variable, and running, reward and pupil size traces as the independent variables (Fig. 3d). Running speed was acquired by a rotary encoder attached to the running wheel. Reward consumption was quantified as the square wave licking signal acquired from the capacitive lick detector convolved with a spline. The spline was picked from a previously used^24^ set of splines, choosing the one that explained the most variance for most cells in the dataset in subsequent regressions. All predictor traces were *z*-scored. Having run the regressions, we removed all the cells for which the model predicted less than 5% of the total variance. For the remaining cells we reran the model with one of the variables removed to estimate their impact on the explained variance. By dividing the full-model *r*^2^ with the *r*^2^ from each of the variable-excluded models, we calculated the percentage contribution of each variable to the variance explained in the full model.

### Fiber photometry

Fiber photometry (Extended Data Fig. 3a–d) was performed as in our previous work^7^. Photometry signals were detrended by fitting a convex hull around the raw photometry trace such that, moving forwards in time, monotonically decreasing vertices were saved into a template. The template was linearly interpolated to match the length of the photometry trace and then subtracted from it. After detrending, all photometry traces were *z*-scored.

### Coherence analysis

Coherence between photometry and pupil dilation was calculated via the multitaper method using custom Python scripts built on the NiTime library (https://nipy.org/nitime/). First, photometry and pupil traces were clipped to the same length (approximately 38 min) across all mice. Coherence at each frequency was calculated from adaptive weighting of the first seven tapers to target a constant resolution bandwidth. Thereby, coherence was computed with an epoch length of the entire trace and then averaged across mice.

### Statistics and reproducibility

Randomization of groups was performed wherever multiple groups of animals or interventions were compared; the experimenter was blinded to group identity during data collection and analyses. Unless otherwise specified, all raw data processing and statistical analysis were done in MATLAB. The statistical tests and their results are shown in the figure legends, along with the *n* values the tests were based on. For the parametric tests, data were tested for normality and equality of variance. *P* < 0.05 is indicated with a single asterisk, and values *P* < 0.01 are indicated with two asterisks, with all values below 0.05 accepted as significant. All error bars show the s.e.m.

### Reporting summary

Further information on research design is available in the Nature Portfolio Reporting Summary linked to this article.

## Online content

Any methods, additional references, Nature Portfolio reporting summaries, source data, extended data, supplementary information, acknowledgements, peer review information; details of author contributions and competing interests; and statements of data and code availability are available at 10.1038/s41593-023-01365-w.

## Supplementary information

Reporting Summary

## Data Availability

Preprocessed data are available at https://osf.io/5dx6u/?view_only=f676e1a52352471a91c9c3585ed11004. Further data are available from the authors upon reasonable request. Source data are provided with this paper.

## Source data

Source Data Fig. 1 Raw source data.
Source Data Fig. 2 Raw source data.
Source Data Fig. 3 Raw source data.
Source Data Extended Data Fig. 1 Raw source data.
Source Data Extended Data Fig. 2 Raw source data.
Source Data Extended Data Fig. 3 Raw source data.
Source Data Extended Data Fig. 4 Raw source data.

## Code availability

The code to analyze the preprocessed data is available at https://osf.io/5dx6u/?view_only=f676e1a52352471a91c9c3585ed11004. Further code is available upon request.
