# Supplementary material for: Control and coding of pupil size by hypothalamic orexin neurons
Source: Nat Neurosci. 2023 Jun 19;26(7):1160–4. doi: 10.1038/s41593-023-01365-w (PMC10322704; doi:10.1038/s41593-023-01365-w)
Supplement: Supplementary file 1 — Reporting Summary [file 41593_2023_1365_MOESM1_ESM.pdf]

nature portfolio

Corresponding author(s): Denis Burdakov

Last updated by author(s): Apr 18, 2023

Reporting Summary

Nature Portfolio wishes to improve the reproducibility of the work that we publish. This form provides structure for consistency and transparency in reporting. For further information on Nature Portfolio policies, see our Editorial Policies and the Editorial Policy Checklist.

Statistics

For all statistical analyses, confirm that the following items are present in the figure legend, table legend, main text, or Methods section.

n/a	Confirmed	
☐	☒	The exact sample size (*n*) for each experimental group/condition, given as a discrete number and unit of measurement
☐	☒	A statement on whether measurements were taken from distinct samples or whether the same sample was measured repeatedly
☐	☒	The statistical test(s) used AND whether they are one- or two-sided<br>*Only common tests should be described solely by name; describe more complex techniques in the Methods section.*
☐	☒	A description of all covariates tested
☐	☒	A description of any assumptions or corrections, such as tests of normality and adjustment for multiple comparisons
☐	☒	A full description of the statistical parameters including central tendency (e.g. means) or other basic estimates (e.g. regression coefficient) AND variation (e.g. standard deviation) or associated estimates of uncertainty (e.g. confidence intervals)
☐	☒	For null hypothesis testing, the test statistic (e.g. *F*, *t*, *r*) with confidence intervals, effect sizes, degrees of freedom and *P* value noted<br>*Give P values as exact values whenever suitable.*
☒	☐	For Bayesian analysis, information on the choice of priors and Markov chain Monte Carlo settings
☒	☐	For hierarchical and complex designs, identification of the appropriate level for tests and full reporting of outcomes
☐	☒	Estimates of effect sizes (e.g. Cohen's *d*, Pearson's *r*), indicating how they were calculated

Our web collection on statistics for biologists contains articles on many of the points above.

Software and code

Policy information about availability of computer code

Data collection	MATLAB 2019a, LabView 2018, Spinnaker SDK
Data analysis	MATLAB 2019a, DeepLabCut 2.1, Python 3.0, ImageJ 15.2

For manuscripts utilizing custom algorithms or software that are central to the research but not yet described in published literature, software must be made available to editors and reviewers. We strongly encourage code deposition in a community repository (e.g. GitHub). See the Nature Portfolio guidelines for submitting code & software for further information.

Data

Policy information about availability of data

All manuscripts must include a data availability statement. This statement should provide the following information, where applicable:

- Accession codes, unique identifiers, or web links for publicly available datasets
- A description of any restrictions on data availability
- For clinical datasets or third party data, please ensure that the statement adheres to our policy

Source Data are provided with this paper and in the online repository, along with preprocessed data at https://osf.io/5dx6u/?view_only=f676e1a52352471a91c9c3585ed11004. Further data is available upon request.

Human research participants

Policy information about studies involving human research participants and Sex and Gender in Research.

Reporting on sex and gender	N/A
Population characteristics	N/A
Recruitment	N/A
Ethics oversight	N/A

Note that full information on the approval of the study protocol must also be provided in the manuscript.

Field-specific reporting

Please select the one below that is the best fit for your research. If you are not sure, read the appropriate sections before making your selection.

☒ Life sciences ☐ Behavioural & social sciences ☐ Ecological, evolutionary & environmental sciences

For a reference copy of the document with all sections, see nature.com/documents/nr-reporting-summary-flat.pdf

Life sciences study design

All studies must disclose on these points even when the disclosure is negative.

Sample size	Sample size was not statistically predetermined, and has instead been chosen such as to be comparable to previous publications: https://www.ncbi.nlm.nih.gov/pmc/articles/PMC4373539/.
Data exclusions	No data were excluded
Replication	Each experiment was done at least in two cohorts, where the results seemed to match up between the cohorts.
Randomization	Randomization of groups was performed wherever multiple groups of animals or interventions were compared and the experimenter was blinded to group identity.
Blinding	Wherever multiple groups of animals or interventions were compared the experimenter was blinded to group identity during data collection and analysis.

Reporting for specific materials, systems and methods

We require information from authors about some types of materials, experimental systems and methods used in many studies. Here, indicate whether each material, system or method listed is relevant to your study. If you are not sure if a list item applies to your research, read the appropriate section before selecting a response.

Materials & experimental systems

n/a	Involved in the study	
☒	☐	Antibodies
☒	☐	Eukaryotic cell lines
☒	☐	Palaeontology and archaeology
☐	☒	Animals and other organisms
☒	☐	Clinical data
☒	☐	Dual use research of concern

Methods

n/a	Involved in the study	
☒	☐	ChIP-seq
☒	☐	Flow cytometry
☒	☐	MRI-based neuroimaging

Animals and other research organisms

Policy information about studies involving animals; ARRIVE guidelines recommended for reporting animal research, and Sex and Gender in Research

Laboratory animals	Study used mice (>8 weeks in age).

Laboratory animals	Strains: C57BL/6, C57BL/6-Tg(Dbh-icre)1Gsc, ORX-DTR (on C57BL/6 background).
Wild animals	Study did not involve wild animals
Reporting on sex	Animals were grouped across sex and information about numbers given in the Methods section. Separate data provided to reviewers
Field-collected samples	Study did not involve field-collected samples.
Ethics oversight	All animal experiments were performed in accordance with the Animal Welfare Ordinance (TSchV 455.1) of the Swiss Federal Food Safety and Veterinary Office, and approved by the Zurich Cantonal Veterinary Office.

Note that full information on the approval of the study protocol must also be provided in the manuscript.
